# Emergence of Online Teaching for Plastic Surgery and the Quest for Best Virtual Conferencing Platform: A Comparative Cohort Study

**DOI:** 10.1055/s-0042-1757334

**Published:** 2023-02-01

**Authors:** Suvashis Dash, Raja Tiwari, Amiteshwar Singh, Maneesh Singhal

**Affiliations:** 1 Department of Plastic, Reconstructive and Burns Surgery, All India Institute of Medical Sciences, New Delhi, India

**Keywords:** residency training, online learning, e-learning, plastic surgery, online platform, Zoom, COVID-19

## Abstract

**Background** As the coronavirus disease 2019 virus made its way throughout the world, there was a complete overhaul of our day-to-day personal and professional lives. All aspects of health care were affected including academics. During the pandemic, teaching opportunities for resident training were drastically reduced. Consequently, medical universities in many parts across the globe implemented online learning, in which students are taught remotely and via digital platforms. Given these developments, evaluating the existing mode of teaching via digital platforms as well as incorporation of new models is critical to improve and implement.

**Methods** We reviewed different online learning platforms used to continue regular academic teaching of the plastic surgery residency curriculum. This study compares the four popular Web conferencing platforms used for online learning and evaluated their suitability for providing plastic surgery education.

**Results** In this study with a response rate of 59.9%, we found a 64% agreement rate to online classes being more convenient than normal classroom teaching.

**Conclusion** Zoom was the most user-friendly, with a simple and intuitive interface that was ideal for online instruction. With a better understanding of factors related to online teaching and learning, we will be able to deliver quality education in residency programs in the future.

## Introduction

We are during a global humanitarian crisis of unprecedented proportions: the deadly second wave of coronavirus disease 2019 (COVID-19), as well as the possibility of a third wave, looming right around the corner. It had brought a complete overhaul of our day-to-day personal and professional lives. 1 All aspects of health care have been affected including the academics such that the teaching opportunities for resident education during the pandemic have reduced drastically. Classroom and bedside teaching, face-to-face workshops, and multidisciplinary discussion meetings have been moved to virtual platforms and this arrangement is expected to stay in the future too.

The pandemic period gave rise to a parallel yet noticeably successful scholastic pandemic throughout the global plastic surgery community—a *webinar pandemic* . 2 Even before COVID-19, there was already high growth and adoption of e-learning technology outside the medical field. But after it, there has been a significant surge in the usage of video conferencing tools. It was challenging for educators and students to familiarize themselves with these platforms. After an initial lag period, we witnessed an exponential rise in Web-based lecture series and case discussions via virtual platforms. It also became difficult to choose the best virtual teaching program for any institution around this time.

Although the platforms have been described there is no study available to find out the best one from a user perspective. 3 These online teaching applications enabled covering of maximum possible portions of residency training curriculum despite curtailment of clinical exposure without the need for unity in time or place.

Many experts, including the authors, were wondering whether the adoption of online learning will continue to persist postpandemic, and how such a shift would impact the delivery of medical education.

## Methods

**Fig. 1:**
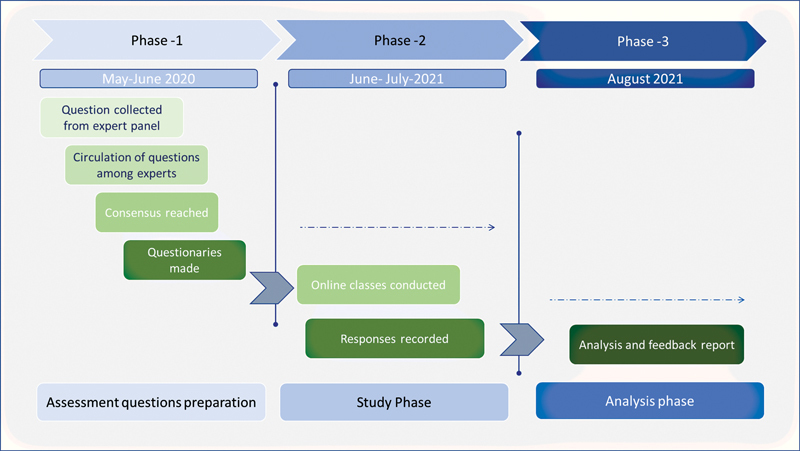
Flowchart for the methodology of the study.

A prospective study was done from May to August 2020 to survey responses from plastic surgery residents from different institutes across the country; conducted the study in three phases ( Fig. 1 ):

Phase 1: Questionnaire preparation and validations.
Phase 2: Response collection.
Phase 3: Analysis.

In the first phase, a panel of experts comprising of seven plastic surgeons, two educationists (an expert specialized in the methods of education and assessment), and one software consultant framed the questionnaires, comprising different aspects of an online training like teaching, content, technicality, and delivery. The questions were circulated in three rounds, among the experts after compilation to take their opinion on the framed questions. Repetitive, ambiguous, and inconsistent questions were removed from the pool. After consensus was reached among the experts the format was accepted for the study (as *supplemental digital content* ). The questionnaires have 40 questions with subsections like demography of the respondent, platform name, course content, teaching mode, usage trends, technical aspects of the platform, the overall experience of the respondent, self-evaluation, plastic surgery content, and suggestions. The responses were in the form of a Likert scale for evaluation.

In the second phase, the questionnaires were converted to an online format in Google Forms (Google LLC, CA). Primarily, the purpose of this study was to understand the attributes of academic teaching and training of plastic surgery residents through an online platform during the COVID-19 pandemic. Second, we have tried to compare four different online platforms to compare their efficacy in teaching plastic surgery academic schedule. The topics for the class were as per standard curriculum of institute and classes were conducted 5 days a week from Monday to Friday. We studied four platforms during this study. In the initial phase, the plan was to identify an appropriate model for teaching so initially we started with the Zoom app (San Jose, CA), then we used Skype (Skype Technologies, Microsoft, WA), then Microsoft Teams (Microsoft), and Google Meet (Google LLC). We have tried 5 days for each application. A weekly theme was followed to allow in-depth discussion of a particular topic. For the evaluation of online teaching, five types of classes were conducted. To avoid bias, similar type of class formats were used for faculty lectures, case presentations, operative technique seminars, didactic lectures, and journal club discussions, each class was scheduled for 90 minutes, in the first half, the presenter talked on the topic using a PowerPoint presentation and in the latter half, the faculty engaged in question-answers using a problem-based approach to highlight the key learning points. Pictographs, video, and live use of white board was used to facilitate detailed teaching of the subject. As online teaching was newly started in the residency curriculum, for quality improvement, at the end of class, feedback was obtained anonymously via Google Forms from all participants. Consent was taken from each person before submitting their responses.

### Statistical Analysis

Data was compiled on MS Excel (Microsoft Inc. US, version 2019) and analyzed using IBM SPSS software (version-20). Categorical variables were expressed as frequency and proportions whereas continuous variables were expressed as mean and standard deviation. One-way analysis of variance was used to compare the mean difference between the groups whereas chi-square test was used to compare proportions. *p* -Value less than 0.05 was considered statistically significant.

## Results

### Demographic Data

**Fig. 2:**
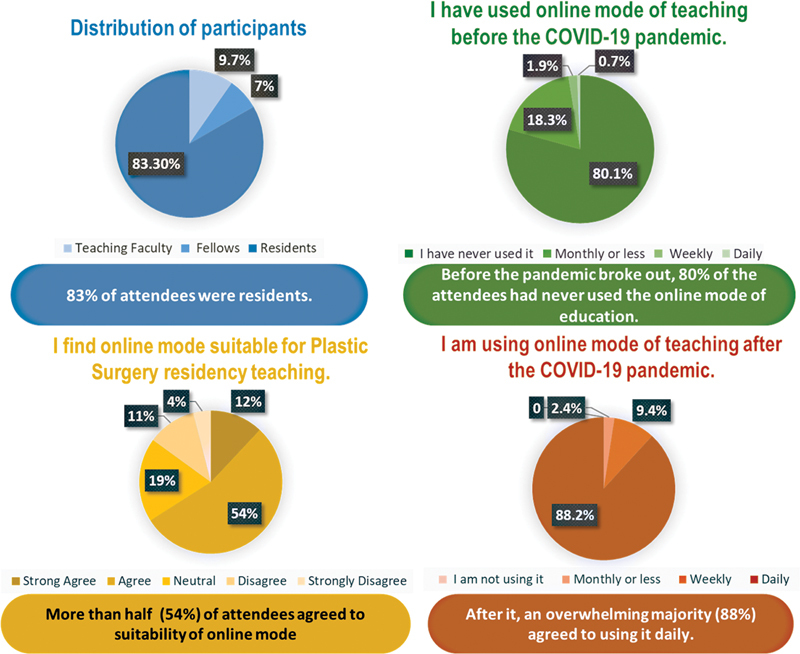
Demographic representation of survey respondents.

A total of 496 attendees took part in this lecture series distributed as five classes per week for a total of 20 classes, and 297 attendees responded to the survey. Therefore, our response rate was 59.9%. Participants were plastic surgery residents, followed by teaching faculty members and fellows ( Fig. 2 ). Out of all participants, 75 (25.3%) participants each used Zoom and Google Meet platform followed by 24.9 and 24.6% participants attending the class using Microsoft Teams and Skype, respectively. Table 1 reveals that the baseline variables were comparable among participants using different platforms ( *p* > 0.05). Most common type of class attended irrespective of platform used was clinical presentation followed by faculty lectures and journal clubs.

**Table 1:** Comparison of baseline variables of classes organized with different platforms

Baseline variables		Zoom ( *n* = 75)	Google Meet ( *n* = 75)	Microsoft Teams ( *n* = 74)	Skype ( *n* = 73)	*p* -Value
Participant age (mean ± SD)		31.04 ± 2.7	31.23 ± 2.55	31.5 ± 4.99	31.4 ± 2.03	0.85
Participant gender	Male	50 (66.7%)	50 (66.7%)	50 (67.6%)	48 (65.8%)	0.99
	Female	25 (33.3%)	25 (33.3%)	24 (32.4%)	25 (34.2%)	
Type of class	Clinical presentation	43 (57.3%)	35 (46.7%)	28 (37.8%)	36 (49.3%)	0.183
	Faculty lectures	8 (10.7%)	13 (17.3%)	20 (27%)	6 (8.2%)	
	Journal clubs	8 (10.7%)	9 (12%)	11 (14.9%)	13 (17.8%)	
	Operative sessions	8 (10.7%)	7 (9.3%)	5 (6.8%)	6 (8.2%)	
	Seminars	8 (10.7%)	11 (14.7%)	10 (13.5%)	12 (16.4%)	

Abbreviation: SD, standard deviation.

### Subcategories Responses

**Fig. 3:**
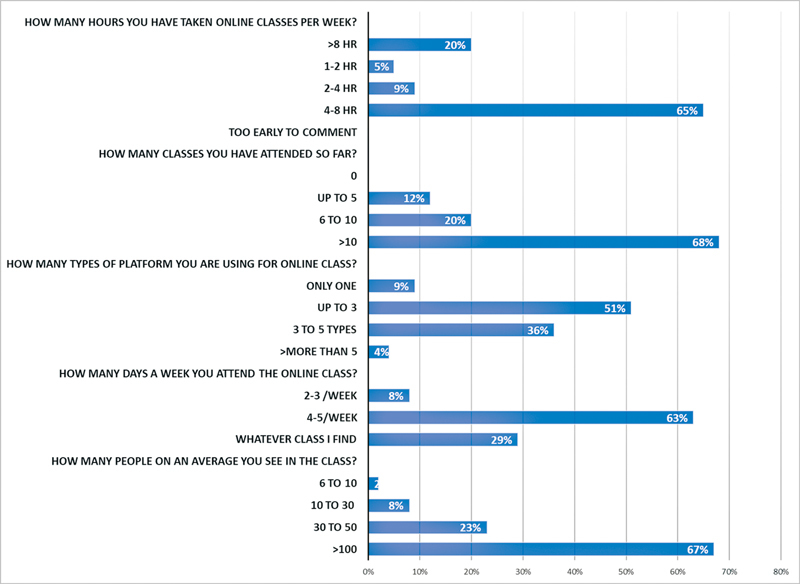
Graphical analysis of usage trends of survey respondents.

A. Course content:
Online platforms were found to facilitate highly effective communication during classes as more than 90% attendees responded either agreed or strongly agreed in this context. While 75% of the participants agreed that the contents were balanced and well-managed and the discussions at the end of each session were productive. Eighty-five percent of participants agreed that the delivery of the content was adequate. Of late, there has been a change in the trend of learning as more residents have moved from offline literature to online resources. As a result, a smooth shift from offline to online classes was possible and 65% of participants agreed that these platforms were suitable to teach the subject. However, 16% disagreed with this, citing difficulty to visualize certain concepts especially related to operative procedures which are clearer during on-table teaching ( Table 2 ).
B. Teaching mode:
We found 87% participants agreed that adequate time was given by the presenter. The participants did not face any problem pertaining to voice modulation and clarity, as 81% agreed to this. We found 74% of attendees responded that the teaching experience via online platforms was close to the normal classroom ( Table 2 ).
C. Usage trends:
Sixty-five percent of participants claimed to be clocking in 4 to 8 hours online per week and an additionally 20% attendees spent more than 8 hours per week. We found 68% reported that they attended more than 10 classes per week distributed over 4 to 5 working days. After travel restrictions, as webinars gained acceptance, many of the previously unknown meeting platforms became popular. About 80% of residents attended webinars on minimum three different platforms. The average attendance in most classes used to be between100 and 130 ( Fig. 3 ).
D. Technical aspects:
The most important technical aspects of online platforms undeniably would be the quality of audio and video transmission. The attentiveness of the audience is largely dependent on these. Among participants using various platforms, technical difficulties were faced in significantly higher proportions of participants using Skype ( *p* < 0.05). Audio quality was comparable in Teams and Zoom, whereas video quality was significantly better in Zoom ( *p* < 0.05). Understanding of overall class as well as experience of class on laptop was significantly better with Zoom platform ( *p* < 0.05). Overall experience was significantly better with Zoom followed by Microsoft Teams and Google Meet ( *p* < 0.05). Technical aspects have been elaborated in Table 3 .
E. Overall experience:
Maximum participants agreed to spend money on buying in case they were given the opportunity (69%, *p* < 0.05). Thoughts of 64% of residents resonated with the idea of the convenience of participating in online classes while another 26% chose to remain neutral stating that a lot depended on the content of the class. About 59% of residents denied difficulty in keeping concentrations during classes ( Table 4 ).
Another aspect of online webinars is that we tend to become self-aware because one can see itself in the video and thus becomes more cautious of how they are looking on camera leading to anxiety and even distraction. However, a mixed response was obtained from residents with 37% agreeing, 29% remaining neutral, and 27% denying becoming self-aware during the class. Despite this, approximately 70% combinedly agreed that they took special care before class to make their surroundings appropriate. Twenty percent who combinedly disagreed, some reported that they preferred using virtual backgrounds ( Table 4 ).
F. Self-evaluation:
For this study, five classes were conducted/week, four of them were presented by residents and one by the faculty. Sixty-three percent of residents gave feedback after the classes to the presenter about the quality of their presentation, 96% respondents combinedly agreed unanimously about the successful learning outcome of these classes which left them well-informed about the clinical conditions. The majority (72%) agreed that the conduct of the classes enabled them to interact with each other. Everyone affirmed that proper discipline and etiquette were followed during the classes ( Table 4 ).
G. Plastic surgery teaching:
  A commonly accepted opinion about interactive sessions being more effective than didactic lectures were confirmed in the study as 92% showed an inclination toward interactive sessions. The online platforms are equipped with one of the most essential features of the soft board, where the presenter could easily illustrate the diagrams creating visual imagery. Ninety-five percent agreed that this enhanced their learning outcome. Screen share was accepted as the paramount feature by all, with 60% of the residents strongly agreeing to it.

**Table 2:** Evaluation of the course content and teaching modality based on survey responses

Survey responses		% of total ( *n* = 297)	*p* -Value
Course content			
Communication during the class was effective	Strongly agree	40 (120)	0.001
	Agree	52 (154)	
	Neutral	3 (9)	
	Disagree	5 (14)	
	Strongly disagree	0 (0)	
Content was balanced and well managed	Strongly agree	23 (69)	0.001
	Agree	52 (153)	
	Neutral	10 (29)	
	Disagree	5 (16)	
	Strongly disagree	10 (30)	
The delivery of the content was adequate	Strongly agree	31 (93)	0.01
	Agree	55 (162)	
	Neutral	7 (22)	
	Disagree	7 (20)	
	Strongly disagree	0 (0)	
Online platform is not suitable for teaching my subject	Strongly agree	2 (7)	0.001
	Agree	14 (42)	
	Neutral	21 (61)	
	Disagree	42 (125)	
	Strongly disagree	21 (62)	
Teaching mode			
Topic of teaching was properly chosen	Strongly agree	33 (99)	0.001
	Agree	58 (172)	
	Neutral	1 (2)	
	Disagree	2 (7)	
	Strongly disagree	6 (17)	
The teaching time was adequate	Strongly agree	18 (52)	0.001
	Agree	69 (204)	
	Neutral	6 (18)	
	Disagree	3 (10)	
	Strongly disagree	4 (13)	
Adequate attention was given to you by the teacher	Strongly agree	33 (97)	0.01
	Agree	54 (159)	
	Neutral	9 (27)	
	Disagree	1 (4)	
	Strongly disagree	3 (10)	
Voice modulation, clarity of teaching was appreciable in online classroom	Strongly agree	11 (34)	0.001
	Agree	70 (207)	
	Neutral	11 (34)	
	Disagree	5 (14)	
	Strongly disagree	3 (8)	
Teaching experience was close to that of normal classroom	Strongly agree	7 (22)	0.001
	Agree	67 (198)	
	Neutral	16 (47)	
	Disagree	7 (20)	
	Strongly disagree	3 (10)	

**Table 3:** Responses about technical aspects of different virtual conferencing platforms

Technical aspects		( *n* = 75)	( *n* = 75)	( *n* = 74)	( *n* = 73)	*p* -Value
Have you faced any difficulties using the online platform?	Strongly agree	0 (0%)	4 (5.3%)	0 (0%)	8 (11%)	0.001
	Agree	15 (20%)	19 (25.3%)	6 (8.1%)	37 (50.7%)	
	Neutral	10 (13.3%)	12 (16%)	10 (13.5%)	7 (9.6%)	
	Disagree	50 (66.7%)	38 (50.7%)	56 (75.7%)	21 (28.8%)	
	Strongly disagree	0 (0%)	2 (2.7%)	2 (2.7%)	0 (0%)	
Audio of this platform was clear	Strongly agree	0 (0%)	0 (0%)	10 0.5%)	0 (0%)	0.001
	Agree	60 (80%)	51 (68%)	62 (83.8%)	37 (50.7%)	
	Neutral	5 (6.7%)	12 (16%)	0 (0%)	10 (13.7%)	
	Disagree	10 (13.3%)	10 (13.3%)	2 (2.7%)	22 (30.1%)	
	Strongly disagree	0 (0%)	2 (2.7%)	0 (0%)	4 (5.5%)	
Video quality of this platform was clear	Strongly agree	10 (13.3%)	20 (26.7%)	14 (18.9%)	9 (12.3%)	0.01
	Agree	65 (86.7%)	43 (57.3%)	54 (73%)	24 (32.9%)	
	Neutral	0 (0%)	4 (5.3%)	6 (8.1%)	17 (23.3%)	
	Disagree	0 (0%)	8 (10.7%)	0 (0%)	19 (26%)	
	Strongly disagree	0 (0%)	0 (0%)	0 (0%)	4 (5.5%)	
Understanding of overall class was different in this platform?	Strongly agree	0 (0%)	2 (2.7%)	6 (8.1%)	0 (0%)	0.001
	Agree	65 (86.7%)	40 (53.3%)	43 (58.1%)	47 (64.4%)	
	Neutral	10 (13.3%)	23 (30.7%)	21 (28.4%)	11 (15.1%)	
	Disagree	0 (0%)	10 (13.3%)	4 (5.4%)	11 (15.1%)	
	Strongly disagree	0 (0%)	0 (0%)	0 (0%)	4 (5.5%)	
Using a laptop for class gives better experience than mobile	Strongly agree	40 (53.3%)	14 (18.7%)	28 (37.8%)	23 (31.5%)	0.001
	Agree	35 (46.7%)	31 (41.3%)	16 (21.6%)	32 (43.8%)	
	Neutral	0 (0%)	28 (37.3%)	26 (35.1%)	14 (19.2%)	
	Disagree	0 (0%)	2 (2.7%)	4 (5.4%)	4 (5.5%)	
	Strongly disagree	0 (0%)	0 (0%)	0 (0%)	0 (0%)	

**Table 4:** Evaluation of the overall experience with online teaching and self-evaluation of participants

Survey responses		% of total ( *n* = 297)	*p* -Value
Overall experience			
Given an opportunity I won't mind spending money for buying an online class platform	Strongly agree	16% (49)	0.001
	Agree	53 (158)	
	Neutral	18 (54)	
	Disagree	5 (16)	
	Strongly disagree	7 (20)	
I find it more convenient compared with the normal class room	Strongly agree	13 (39)	0.001
	Agree	51 (151)	
	Neutral	26 (77)	
	Disagree	6 (18)	
	Strongly disagree	4 (12)	
The most difficult part for me is to keep my concentration during the online class	Strongly agree	3 (8)	0.01
	Agree	14 (43)	
	Neutral	11 (33)	
	Disagree	59 (174)	
	Strongly disagree	13 (39)	
I became self-aware during the class	Strongly agree	4 (12)	0.001
	Agree	37 (110)	
	Neutral	29 (85)	
	Disagree	27 (81)	
	Strongly disagree	3 (9)	
I take special care before class to make appropriate surrounding	Strongly agree	13 (39)	0.001
	Agree	57 (168)	
	Neutral	11 (32)	
	Disagree	12 (37)	
	Strongly disagree	7 (21)	
Self-evaluation			
I have actively contributed to the online teaching	Strongly agree	13 (37)	0.001
	Agree	52 (153)	
	Neutral	29 (85)	
	Disagree	5 (16)	
	Strongly disagree	1 (4)	
I have given feedback to the presenter after the class	Strongly agree	16 (47)	0.001
	Agree	63 (188)	
	Neutral	12 (35)	
	Disagree	9 (27)	
	Strongly disagree	0 (0)	
I think I have achieved good learning outcome by the online class room	Strongly agree	16 (49)	0.454
	Agree	79 (236)	
	Neutral	4 (12)	
	Disagree	0 (0)	
	Strongly disagree	0 (0)	
I have interacted with other students during class	Strongly agree	19 (55)	0.001
	Agree	55 (163)	
	Neutral	14 (42)	
	Disagree	10 (29)	
	Strongly disagree	3 (8)	
I follow online etiquettes and discipline during the class	Strongly agree	48 (143)	0.01
	Agree	51 (152)	
	Neutral	1 (2)	
	Disagree	0 (0)	
	Strongly disagree	0 (0)	

## Discussion

With the world rapidly becoming digital, people now rely on Web-conferencing far more than ever before, these platforms enabled businesses and educational institutions to continue being productive through these challenging times.

While some believe that the impromptu move to online learning, with no training, variable access to the Internet, and extemporaneous implementation will result in a poor user experience and will lack sustainability. Others believe that a new hybrid model of education will emerge, with significant benefits. 4

The big question that arises is “ *Whether online learning is better than or at least as effective as classroom teaching?* ” The answer is highly debatable as for some, online classes are more appropriate while for others classroom teaching is a preferred delivery method. Some authors have tried to resolve the issue through a systematic review and meta-analysis which reports that there is no evidence that classroom learning works better. Compared with offline learning, online learning has advantages to enhance undergraduates' knowledge and skills. 4

On the contrary, few studies have reported negative aspects of online learning. 5 6 Another approach for health profession education that has grown rapidly is blended learning, and it has been consistently shown to be an optimum method for knowledge acquisition. 7

Online learning is described as Internet-based learning and is considered a more recent version of distance learning. 8 Over the last decade, medical schools have been slowly adapting it into pedagogical methods and coronavirus was just the right catalyst for its widespread acceptance. After the announcement of the lockdown, many national and international plastic surgery residency programs and societies took initiative in organizing webinar series for teaching. It was not at all surprising to note from our study that before the pandemic 80% of the attendees had never used the online mode of education and after it, 88% agreed to have used it. This drastic turnaround can be attributed to the lack of adoption of online learning in India compared with western nations. At this moment, numerous webinars have augmented the number of plastic surgery residents using online platforms. 9

In our study each class was structured according to the “ *Flipped Classroom* ” model, which is the practice of assigning learners didactic material, traditionally covered in lectures, to be learned before class while using face-to-face time for more engaged and active learning. It has been shown to have a positive effect on active learning, 10 and on providing more opportunities for students to engage in critical thinking. It promotes independent learning, and effective interaction with, and learning from their peers 11 and teachers. 12 Using this model, we found highly effective communication during classes as more than 90% agreed positively.

The other aspect that determines the successful delivery of content during online classes is the educator's dialogue delivery and their effort to bridge the “transactional distance.” 13 According to Moore, 14 transactional distance is a social, psychological, and relational distance between teachers and learners that is fluid and manageable based on a function of dialogue and structure. Appropriate voice modulation invokes the cognitive and social presence of the attendees in online medium. 15 “Cognitive presence” indicates the degree to which the learners can understand during exchanges of thoughts—questioning, answering, brainstorming, discussing, and solving a problem. “Social presence” can be achieved when learners project their personal feelings, emotions, questions, and characteristics with the group. 15 16 It will promote meaningful outcomes by encouraging discussions and collaboration within the group. However, we found a minor 10% disagreement on the effectiveness of online classes was observed mainly in the context of operative technique seminars. It was probably due to lack of previous experience of residents on the procedures or due to lag in videos unlike played in real-time.

In our study, high usage of online classes to the tune of 4 to 8 hours online/week was reported by 60% of attendees. This was mainly due to many societies and institutions extending their invites beyond their group for the benefit of residents.

We used the four most popular in our region: Microsoft Teams, Skype, Google Meet, and Zoom. We found Zoom provided the smoothest experience evidenced by the fact only 8% experienced problems with it. It continues to be the go-to video conferencing application despite having security flaws like “Zoom-bombing.” It gives users a simple and clean user interface with solid collaboration tools and a wide range of control over their video experience. Zoom provides a quality interactive interface with feature like nonverbal feedback, polling, breakout rooms, end of meeting feedback, survey, and note taking. Its limited complimentary use also adds it popularity. Microsoft Teams is the next name that comes to mind because of its acclaimed pedigree but it loses to others because of its complex interface. Users reported taking some time to get familiar with where all buttons and options were which did get annoying sometimes especially for not-so-tech-savvy faculty members. Although audio and video issues were common to all platforms, approximately 50% reported facing more problems with Skype. For example, some users faced spontaneous call drops and others complained about disturbance from notification of the snapshot feature. Like Zoom Meetings, Google Meet delivered a clear, consistent, and reliable video experience, but it lost out due to its limited features in the free version for users such as the record meeting feature.

Many attendees agreed that online lectures have many advantages over offline classrooms: (1) opportunity to listen from international faculties/eminent persons whom they can only meet at conferences, (2) gain insights and different perspectives of surgical practice around the world, (3) ability to freely join or leave lectures, (4) chance to record and review lectures later, and (5) no traveling cost and the convenience of attending and presenting from home. 17

The major concerns for online learning are lack of hands-on opportunities and meeting colleagues in person. Virtual cannot replicate the operation theater and clinical experience which is critical for the surgical training. However, in future with advent for high-speed Internet this will be not far, where tele or remote surgery conferences/workshops can be done effortlessly. The feel of being in the operating room, doing preoperative planning, and providing postoperative care to patients can never be taught by prerecorded operative videos. The respondents felt that these platforms do not provide the same level of interaction as in-person meeting. There are also concerns of viewers taking screenshots or recording lectures with patient's clinical photographs and this may not be Health Insurance Portability and Accountability Act compliant. Also, technical glitches and Internet speed are troublesome.

The practice of surgery is becoming increasingly more complex. Ninety-two percent of attendees showed an inclination toward interactive sessions and feel that this technology will help bring a balance between clinical, hands-on education, and didactic lectures. It will enable the continuation of learning from the comfort of time and place, making the best use resources to maximize resident education. Simultaneously, surgical educators need to use innovations to make the best use of data and knowledge to train the next generations of plastic surgeons. 18 The authors believe that this unprecedented change in medical education due to the COVID-19 pandemic has led to the emergence of a new principles of learning.

In the post-COVID time, when physical meeting will be possible even then, the virtual conferencing platforms, may still find utility. Many speculate it will become a norm to include the virtual platforms along with physical meetings to make it a hybrid event. It will be balance between the physical and virtual world. In future with addition of technology like augmented reality, integrated voice assistants will be a value addition to these platforms to make it more productive and interesting. 19 The cost effectiveness of virtual meetings, generating more audience, and ease of joining from any place with at par productivity are greatest advantages. The platforms will continue to be used by health care workers for telemedicine purposes. 20 Live language translation into multiple languages simultaneously in the future may again prove to be of utility to include participation from a wider population.

This is a well-designed study with good sample size, describing different aspects of online teaching, and comparing various platforms. The study was limited to four platforms, missed some of the popular platforms, and several new updates were added to different platforms subsequently which were not available at the time of study are limitations of the study.

Video conferencing software enabled many businesses to continue to function and educational institutions to keep teaching, during times of social distancing. Plastic surgery community should take this opportunity to analyze and inculcate online learning to overcome the challenges plaguing the current residency education system. Based on this study online learning is equally effective compared with classroom teaching, therefore a method of blended learning shall prove to be a reliable platform even after the pandemic is over and social distancing is not necessary. Plastic surgery is such a diverse field that there is a plethora of information available to learn, and therefore may be the most suitable subject where remotely guided self-directed learning can enhance the level of competency of future residents. Although by now most institutions have their favorite platform, based on this study, Zoom was identified as the quintessential tool for continuing online medical education.
