# Supplementary material for: Emergence of Online Teaching for Plastic Surgery and the Quest for Best Virtual Conferencing Platform: A Comparative Cohort Study
Source: Arch Plast Surg. 2023 Feb 1;50(2):200–9. doi: 10.1055/s-0042-1757334 (PMC10049808; doi:10.1055/s-0042-1757334)
Supplement: Supplementary file 1 — Supplementary Material [file 10-1055-s-0042-1757334-s22feb0021cme.pdf]

Anonymous response collection

Subject name- Date-

Platform- Age Sex

Zoom	Microsoft Team	Google meet	Skype	Others

A. Course content

1	What are types of class you attend via online platform	Faculty lectures	Clinical presentation	JC	Operative plans	seminars
2	Effective communication during the class is possible	Strongly agree	agree	neutral	disagree	Strongly disagree
3	Content was balanced and well managed	Strongly agree	agree	neutral	disagree	Strongly disagree
4	The delivery of the content was adequate	Strongly agree	agree	neutral	disagree	Strongly disagree
5	Online platform is not suitable for teaching my subject	Strongly agree	agree	neutral	Disagree	Strongly disagree

B. Teaching Mode

1.	Topic of teaching was properly chosen	Strongly agree	agree	neutral	disagree	Strongly disagree
2.	The teaching time was adequate	Strongly agree	agree	neutral	disagree	Strongly disagree
3	Adequate attention was given to you by teacher	Strongly agree	agree	neutral	disagree	Strongly disagree
4	Voice modulation, clarity of teaching was appreciable in online classroom	Strongly agree	agree	neutral	Disagree	Strongly disagree
5	Teaching experience was as close to normal class room	Strongly agree	agree	neutral	Disagree	Strongly disagree

C. Teaching Numbers

1.	How many hours you have taken online classes per week	1-2 hr	2-4 hr	4-8 hr	>8 hr	
2.	How many classes you have attended so far	0	Up to 5	6-10	>10	
3	how many types of platform you are using for online class?	Only one	Up to 3	3-5	>more	
4	How many days a week you attend the class	Once a week	2-3 /week	4-5/week	Whatever class I find	
5	How many people on an average you see in the class?	Up to 5	6-10	1-30	30-50	100

D. Technical aspects

1	Have you faced any difficulties using the platform	Strongly agree	agree	neutral	disagree	Strongly disagree
2	Audio of this platform was clear	Strongly agree	agree	neutral	disagree	Strongly disagree
3	Video quality of this platform was clear	Strongly agree	agree	neutral	disagree	Strongly disagree
4	Understanding of overall class was different in this platform?	Strongly agree	agree	neutral	disagree	Strongly disagree
5	Using a laptop for class gives better experience than mobile devices	Strongly agree	agree	neutral	Disagree	Strongly disagree

 © 2023. The Korean Society of Plastic and Reconstructive Surgeons. All rights reserved.

E. Overall experience

1	Given opportunity i won'o;t mind spending money for the online class platform	Strongly agree	agree	neutral	disagree	Strongly disagree
2	I find it more convenient compared to the normal class room	Strongly agree	agree	neutral	disagree	Strongly disagree
3	The Most difficult part for me, is to keep my concentration during the class.	Strongly agree	agree	neutral	disagree	Strongly disagree
4	I became self-aware during the class	Strongly agree	agree	neutral	disagree	Strongly disagree
5	I take special care to before class to make appropriate surrounding	Strongly agree	agree	neutral	Disagree	Strongly disagree

F. Self-evaluation

1	I have contributed in the teaching actively	Strongly agree	agree	neutral	disagree	Strongly disagree
2	I have given feedback to the presenter of the class.	Strongly agree	agree	neutral	disagree	Strongly disagree
3	I think I have achieved good learning outcome by the online class room	Strongly agree	agree	neutral	disagree	Strongly disagree
4	I have interacted with other students during class.	Strongly agree	agree	neutral	disagree	Strongly disagree
5	I follow Online Etiquettes and discipline during the class.	Strongly agree	agree	neutral	Disagree	Strongly disagree

F. Plastic surgery

1	Interactive session is more important than to didactic lectures.	Strongly agree	agree	neutral	disagree	Strongly disagree
2	Diagrams made during teaching helped me to understand the concepts better.	Strongly agree	agree	neutral	disagree	Strongly disagree
3	Screen sharing aspect is very important to convey the idea.	Strongly agree	agree	neutral	disagree	Strongly disagree
4	3D models, Simulations are needed in future for better understanding of subject	Strongly agree	agree	neutral	disagree	Strongly disagree
5	Suggestion from you to make the experience better					

 © 2023. The Korean Society of Plastic and Reconstructive Surgeons. All rights reserved.
